# Magnitude and factors associated with adherence to Iron and folic acid supplementation among pregnant women in Aykel town, Northwest Ethiopia

**DOI:** 10.1186/s12884-019-2422-4

**Published:** 2019-08-14

**Authors:** Habtamu Assefa, Solomon Mekonnen Abebe, Mekonnen Sisay

**Affiliations:** 1 Gorgora Health Center, University of Gondar, Gondar, Ethiopia; 2 Department of Human Nutrition, Institute of Public Health, College of Medicine and Health Sciences, University of Gondar, Gondar, Ethiopia

**Keywords:** Adherence, Iron and folic acid supplementation, Associated factors, Pregnant women, Ethiopia

## Abstract

**Introduction:**

Physiological changes during pregnancy, foetal growth and development increase the requirement for Iron and Folic Acid. The increased demand of these nutrients is not meet through diet alone due to decreased bioavailability of nutrients during pregnancy. In 2004, Ethiopia adopted the global Iron and Folic Acid supplementation strategy targeting to reduce the prevalence of Iron deficiency anemia in women of reproductive age and children under five, by one third. However, the prevalence of anemia remains high and only 5% of pregnant women took Iron and Folic Acid tablets for 90 days or more during their most recent pregnancy in Ethiopia. Therefore, we conducted this study to assess the magnitude and associated factors of adherence to Iron and Folic Acid supplementation during pregnancy.

**Methods:**

A facility based cross sectional study was conducted from February to May, 2018 among pregnant women in Northwest Ethiopia. Systematic random sampling technique was used to select 418 study subjects. Bivariable and multivariable logistic regression analyses were computed to identify predictor variables.

**Results:**

Rate of adherence to Iron and Folic Acid supplementation among pregnant women was 47.6%. Pregnant women who had a past history of abortion, knowledge of anemia and received health education were more likely to be adherent with Iron and Folic Acid supplementation. Furthermore, knowledge about benefits of the supplement and not facing any problem in the health facilities during Iron and Folic Acid tablet collection were factors associated with Iron and Folic Acid supplementation adherence.

**Conclusions:**

Rate of adherence to Iron and Folic Acid supplementation was low in Aykel town. Therefore, strengthening and promoting health education, creating awareness and avoiding discouraging conditions in health facilities during tablet collection are very crucial to improve and increase the low rate of Iron and Folic Acid supplementation adherence in the study area.

## Background

Physiological changes during pregnancy, foetal growth and development increase the requirement for Iron and Folic Acid. The increased demand for these nutrients is not meet through diet alone due to decreased bioavailability of nutrients among pregnant women. The likelihood of presenting Iron deficiency and Folate deficiency is high if diet is not supplemented with Iron and Folic Acid tablets during pregnancy [1]. Pregnant women are among the risk groups for anemia due to low Iron stores in their body. This is supported by reports from the World Health Organization (WHO), indicating that anemia affected 38.2% of pregnant women globally and 46.3% in African [2]. Concerning the prevalence of anemia among Ethiopian reproductive age women, it is estimated that 24% of them are anemic [3].

In order to reduce the risk of maternal Iron-deficiency anemia, the WHO recommended a daily oral dose of 60 mg Iron and 400 μg Folic Acid (IFA) supplements throughout pregnancy, to begin as early as possible as a routine part of antenatal care [4, 5]. Several studies have reported that the use of any antenatal Iron and Folic Acid supplementation during pregnancy reduces the risk of early neonatal and childhood mortality by preventing maternal anemia, low birth weight, and preterm delivery [6].

Even though, WHO recommends giving all pregnant women a standard dose of 60 mg Iron and 400 μg Folic Acid supplementation for 6 months on a daily basis to prevent maternal anemia and neonatal neural tube defects [4], adherence to IFA supplement during pregnancy is poor and has not improved significantly in the last decades among low and middle income countries. A study done in South Australia showed that 23% of pregnant mothers adhered to IFA supplementation [7]. In Malawi 37% of the pregnant mothers consumed the ideal minimum of 180 IFA tablets [8, 9]. Another study done at the University of Gondar, in Northwest, Ethiopia, reported that 55% of pregnant women adhered to the recommended Iron and Folic Acid supplementation [9].

Lack of Iron and Folic Acid (IFA) supplementation and poor adherence to the supplement during pregnancy is associated with anaemia. Maternal anemia is also associated with low weight gain, congestive heart failure, preterm labour, bleeding, lower resistance to infection, poor cognitive development and reduced work capacity. Likewise, Folic Acid deficiency during pregnancy is also associated with increased risk of neural tube defect, preeclampsia, foetal malformations and preterm delivery [10].

In 2004, Ethiopia adopted the global Iron and Folic Acid supplementation targeting to reduce the prevalence of Iron deficiency anemia in women of reproductive age and children under five, by one third which is expected to be achieved through distributing IFA supplement during ANC visits [11]. However, increases were observed in anemia prevalence from 17 to 24% in the last 5 years among women and the coverage of IFA supplementation during pregnancy has improved from 1 to 5%, but remains at substandard level as only 5% of pregnant women took Iron and Folic Acid tablets for 90 days or more during their most recent pregnancy in Ethiopia [3].

Reasons for poor adherence to IFA supplementation arises from pregnant women’s behaviour such as misunderstanding of instructions, side effects, frustration about the frequency and number of pills taken, nausea and constipation which might make the intervention inadequate to reduce anaemia among pregnant women [4].

Taking in to account the existing problem under study which is a public health problem in Ethiopia, it is believed that this work will provide up-to-date information with regards to the magnitude of IFA adherence and factors influencing it among the study population. In addition, this work identified some contributing factors of adherence which were not captured by the available literatures.

Therefore, the aim of the study was to assess magnitude of adherence and associated factors of Iron and Folic Acid supplementation among pregnant women attending ANC in Aykel town administration public health facilities, Northwest Ethiopia, 2018.

## Methods

### Study setting

The study was conducted in Aykel town, Northwest Ethiopia. The town has a total of eight kebeles. The town is located about 780 km from Addis Ababa, the capital city of Ethiopia. The town population was 53,581 of whom 25,171 were males and 28,406 were females respectively [12]. There were 1350 pregnant women in the town. The town had one primary hospital, one health centre, nine private pharmacies and ten private clinics which are providing health care services for the town and nearby communities [13] .

### Study design and population

A facility based cross sectional study was employed in Aykel town health centers and a primary hospital. The source population was pregnant women who attended antenatal care clinics and were supplemented with Iron and Folic Acid tablets. Pregnant women visiting health facilities at least for a second antenatal visit and received IFA supplementation tablets for at least 1 month prior to data collection period were included in this study. An interview was conducted on selected pregnant women just before receiving ANC services.

### Sample size determination and sampling procedure

The required sample size for this study was determined by using single population proportion estimation formula and considering the following assumptions; the rate of adherence to Iron and Folic Acid supplementation among pregnant women was taken as 55.5% [14], 95% confidence interval and 5% acceptable margin of error. The calculated sample size provided a total of 380 pregnant women. Finally, considering a 10% non-response rate the final sample size become 418. After proportional allocation of pregnant women for the two health facilities, a systematic random sampling technique was employed to include a sample of 418 participants. Sampling fraction (K) was; N/*n* = 890 /418 = 2. Then, the lottery method was employed to identify the first pregnant women to be interviewed and 2 was drawn as the first pregnant woman on whom the interview was started. Consequently, women were identified and an interview was held in every two intervals.

### Data collection tools and procedures

Data was collected using an interviewer administered structured questionnaire. The questionnaire was prepared in the English language and then translated to the local language, Amharic by language expert (who is fluent both in English and Amharic languages). The principal investigator was responsible to oversee and supervise closely the overall data collection activities on a daily basis. Pre-test of the questionnaire was done on 21 pregnant women (5% of the total samples) outside the study area. Following the results of the pre-test, necessary corrections and amendments were made on the questionnaire before the actual data collection was initiated and acceptability and applicability of data collection procedures and tools were also evaluated. Six diploma graduate clinical nurses were recruited as data collectors and supervised by three-degree graduate clinical nurses after providing a 1 day-training to both of them. During home visits, if the caregiver was absent, a second visit was made.

### Measurement of variables

For the current study, the dependent variable was adherence to IFA supplementation. Socio-demographic factors, obstetric and health related factors, supplement related factors, women’s awareness and health service related factors were independent variables of the study.

### Adherence to IFAS

#### Self-report adherence

Pregnant women were asked to report how many times IFA supplementation tablets were taken per week by them from all prescribed tablets in the previous 1 month. In this case pregnant women were considered as adherent to IFA supplementation if they were able to take at least 4 IFA tablets per week in the previous 1 month preceding the survey, otherwise; they were classified as non- adherent to IFA supplementation [15].

#### Morisky-medication adherence score (MMAS Version_8)

Pregnant women adherence label was classified as high, moderate and low if they scored 8, 6–7 and less than or equal to 5 out of 8 questions offered to them to assess label of adherence to IFA supplementation respectively [16, 17]. The English version of the measurement scale was translated to the local language, by a language expert at the Department of English language and literature, School of Social Sciences and Humanities, University of Gondar, Ethiopia (who is fluent both in English and Amharic languages).

#### Knowledgeable about anaemia

Respondents were asked questions related to the cause, signs and symptoms, method of prevention, consequence of anemia and risk groups for anaemia. Accordingly, pregnant women who scored greater than the mean value of correct responses were considered as knowledgeable about anemia otherwise, they were considered as not knowledgeable.

#### Knowledge about benefits of IFAS

To assess pregnant women’s knowledge about the benefits of IFA supplementation respondents were asked questions related to IFA benefits. Meanwhile, pregnant women were classified as knowledgeable and not knowledgeable about the benefits of IFA supplementation if they scored greater than or equal to the mean value and less than the mean value of correct responses respectively.

### Data quality control

To maintain the quality of data the data collection tool was developed carefully with the involvement of all authors and revisions were also made. Language expert who is fluent both in English and Amharic languages has translated the tool. The instrument was pretested in similar communities out of the study area before the actual data collection was started. Both data collectors and supervisors had been trained for 1 day on survey objectives, survey methodology, ethics and basic interview techniques. The principal investigator, all data collectors and supervisors conducted the pre-test. In addition, close daily field supervision was carried out during data collection to monitor the performance of data collectors and to deliver immediate correction/feedback on mistakes noted. The principal investigator and supervisors were responsible to review and check the questionnaire to ensure its consistency and completeness before data collectors left the household on daily basis.

### Data processing and analysis

After the data were checked for its accuracy and completeness data was coded and entered into Epi info version 7, then exported into the Statistical Package for Social Sciences (SPSS) version 22.0 for analysis. Descriptive statistics were used to summarize the variables. Figures and tables were also employed to summarize frequencies and percentages of the variables. Wealth status of the household was analysed by principal component analysis (PCA).

Both bivariable and multivariable logistic regression analysis were computed to identify factors associated with IFA supplementation. Variables with a *p* value of < 0.2 during a bivariable analysis were incorporated into the multivariable logistic regression to control the possible effects of confounders. Adjusted odds ratio (AOR) with corresponding 95% confidence interval (CI) were computed to see the strength of the association and a *p* value of < 0.05 was considered statistically significant. In addition, the Hosmer and Lemeshow test was utilized to test the goodness-of-fit of the final logistic regression model and provided a *p*-value of 0.37.

## Results

### Socio-demographic, obstetric, health and health facility related characteristics of respondents

Four hundred twelve pregnant women were participated in this study. The mean ± standard deviation (SD) age of respondents was 26.8 ± 5.25 years. Most of the study participants, (63.8%), were in the age group of 20–29 years. Whereas 91.75%% of the respondents were Orthodox Christian followers. More than half (58.5%) of the participants were living in the Urban Kebels. Of all mothers 27.67% had attained diploma and above education and only 21.36% had completed secondary education. Almost all (98.3%) of the respondents were married.

Regarding to obstetric history, majority (46.6%) of pregnant women were gravida 2–4 and para 1 (34.71%). More than three fourth (80.34%) of the respondents were in their third trimester. Concerning their time of registration for ANC follow up, more than half of the pregnant women registered early or at less than 16 weeks of gestational age and among all who started ANC follow up only 13.8% had 4 or more ANC visits (Table 1).

**Table 1:** Socio-demographic, obstetric, health and health facility related characteristics of respondents attending antenatal care clinics in Aykel town, Northwest Ethiopia, 2018 (*n* = 412)

Variables	Frequency	Percent (%)
Age		
15–19	22	5.34
20–29	263	63.83
30+	127	30.9
Religion		
Orthodox	378	91.75
Muslim	34	8.25
Residence		
Urban	241	58.50
Rural	171	41.50
Maternal Education		
Cannot read and write	118	28.64
Primary education	92	22.33
Secondary education	88	21.36
Diploma and above	114	27.67
Occupation		
House wife	251	60.92
Government employee	109	26.46
Private employee	52	12.62
Marital status		
Married	405	98.30
Single	7	1.70
Husband education		
Cannot read and write	91	22.09
Primary education	135	32.77
Secondary education	57	13.83
Diploma and above	129	31.31
Family size		
1–3	265	64.32
4–6	103	25.00
7+	44	10.68
Wealth quintiles		
Low	185	44.91
Middle	145	35.19
Highest	82	19.90
Trimester of pregnancy		
Second	81	19.66
Third	331	80.34
Time of registration for ANC^a^ services		
< 16 Week (early)	249	60.44
> 16 week (21)	163	39.56
Number of ANC visits		
≤ 4 visit	355	86.17
> 4 visit	57	13.83
History of abortion during last pregnancy		
Yes	51	20.16
No	202	79.84
Place of delivery for the last child		
Health institution	123	53.50
Home	107	46.50
Number of neonate delivered during last delivery		
One	225	96.57
Two	5	2.15
Three	3	1.29
Problem/s faced during last child delivery		
Yes	56	24.03
No	177	75.97
History of anaemia during your last pregnancy		
Yes	69	16.75
No	180	43.69
History of anaemia during your current pregnancy		
Yes	140	33.98
No	272	66.02
Health education given about supplements in the ANC clinic		
Yes	121	29.37
No	291	70.63
Problem faced in HF^b^ during collecting IFAS^c^		
Yes	66	16.02
No	346	83.98
Distance from home to health facility		
> 30 Minute	193	46.84
< 30 Minute	219	53.16

^a^Antenatal Care

^b^Health facility

^c^Iron and Folic acid supplementation

### Knowledge of pregnant women about anaemia and benefits of Iron and folic acid supplementation during pregnancy

Less than half (45.1%) of them were knowledgeable about anaemia during pregnancy. Respondents knowledge about anaemia was determined by summing up all correct responses provided to them. The mean value of pregnant women knowledge on anemia during pregnancy was 5.41. Concerning their knowledge about benefits of IFA supplementation during pregnancy, more than half (63.8) of them were knowledgeable.

### Adherence to Iron and folic acid supplementation

Based on self-reported adherence, nearly half (47.6%) of pregnant women were adherent to IFA supplementation. Meaning, among all participants of the study, 47.6% of pregnant women had taken 4 or more tablets of IFA supplements per week in the past 1 month preceding the survey. Conversely, more than half (52.4%) of them were non-adherent to IFA supplementation. Measurement of IFA supplementation adherence with Morisky medication adherence score (MMAS Version_8) also showed that 14.1, 33 and 52.9% of pregnant women had high, moderate and low adherence respectively. There was no statistical significant relationship between self-reporting adherence and Morisky medication adherence score (MMAS Version_8). The major reasons mentioned by participants to be adhered to the supplement were knowledge about the health value of IFAS, fear of falling sick and the desire to put the health worker’s advice in to practice. On the other hand, forgetfulness, fear of side effects, and failure to get adequate supplements in the health facility were the primary underlying reasons for the non-adherence to IFA supplementation.

### Factors associated with adherence to Iron and folic acid supplementation

In both bivariable and multivariable analysis, having a history of abortion, knowledge about anaemia, knowledge about benefits of IFA supplementation, health education received about the supplement during ANC visits, not facing any problem in the health institution during collection of IFA tablets were found to be significantly associated with adherence to IFA supplementation after adjusting and controlling for all other variables at *P* value of < 0.05.

Accordingly, pregnant women who didn’t face problems in the health facility were 4.78 times more likely to be adherent to IFA supplementation in comparison to those who did face problems [Adjusted Odds Ratio (AOR) = 4.78, 95% Confidence Interval (CI) = (1.98–11.51)]. Likewise, pregnant women having previous history of abortion were more likely to take four or more IFA tablets [AOR = 3.92, 95% CI = (1.77–8.70)]. Moreover, receiving information about the importance of IFA supplementation increases the odds of adherence [AOR = 3.72, 95% CI = (1.80–1.71)]. A high likelihood of being adhered were also observed among pregnant women who had knowledge about the benefits of IFA supplementation [AOR = 3.56, 95% CI = (1.77, 7.14)] and being knowledgeable about anemia during pregnancy [AOR = 2.30, 95% CI = (1.15–4.59)] (Table 2).

**Table 2:** Factors Associated with Adherence to Iron and Folic Acid Supplementation among pregnant women attending antenatal care clinics in Aykel town, Northwest Ethiopia, 2018 (*n* = 412)

Variables	Adherence		COR (95%CI)	AOR (95%CI)	*P*-value
	Yes, no (%)	No, no (%)			
Number of Children					
0	82 (44.5)	102 (55.5)	1.38 (0.67–2.83)	1.07 (0.31, 3.68)	0.657
1–3	138 (52.0)	127 (47.9)	2.13 (1.01–4.5)	1.2 (0.33, 4.42)	0.594
4–6	29 (46.7)	33 (53.3)	1.5 (0.66–3.4)	0.86 (0.20, 3.62)	0.401
≥ 7	14 (36.8)	24 (63.2)	1	1	
History of abortion					
Yes	36 (70.5)	15 (29.4)	2.78 (1.39, 5.24)	3.92 (1.77, 8.7)	0.003
No	95 (47.1)	107(52.9)	1	1	
Knowledge about anemia					
Knowledgeable	130 (70.0)	56 (30.0)	5.62 (3.68, 8.6)	2.3 (1.15, 4.59)	0.017
Not knowledgeable	66 (29.0)	160 (71.0)	1	1	
Knowledge of IFAS^a^					
knowledgeable	159 (60.5)	104 (39.5)	4.62 (2.96, 7.23)	3.56 (1.77, 7.14)	0.002
Not knowledgeable	37(24.9)	112 (75.1)	1	1	
HE given about IFAS^a^					
Yes	9 5 (78.5)	26 (21.5)	6.83 (4.16, 1.23)	3.72 (1.8, 7.71)	0.001
No	101 (34.8)	189 (65.2)	1	1	
Problem faced in HF^b^					
Yes	17 (25.7)	49 (74.3)	1	1	
No	179 (51.7)	167(48.2)	3.08 (1.71, 5.57)	4.78 (1.98, 11.51)	0.001
Distance to health institutions					
> 30 min	77 (40)	116 (60)	1	1	
< 30 min	119 (54.3)	100 (45.6)	1.79 (1.21,2.65)	1.32(0.68, 2.55)	0.45

^a^Iron and Folic acid supplementation

^b^Health facility

## Discussion

In the present study, 47.6% of pregnant women adhered to IFA supplementation. This finding is in line with a study conducted in Debre Markos town, Ethiopia (55.5%) [14]. However, this figure was lower than studies done in Assela town, Ethiopia (59.8%), Eritrean refugee camps (64.7%) and Mizan Aman town, Ethiopia (70.6%) [18–20]. The difference between the current and previous study findings may be explained by the difference in study areas, living conditions and socio cultural differences. In addition, this might be related to lower rate of pregnant women literacy in the current study setting (27.67%). Educated women can understand the messages passed from health professionals, able to read, put in to practice and tend to consume at least 90 IFA tablets during pregnancy as compared to uneducated women [21].

Again the findings of the current study is not in agreement with studies conducted in different regions of Ethiopia; such as in Afar region (22.9%), Goba district (18%) and Misha district (39.2%) [22–24]. This discrepancy may be attributed to sample size and socio economic, cultural, health seeking behaviour, study period gap and geographical difference across the study population.

According to the present study finding history of abortion showed a significant association with Iron and Folic Acid supplementation adherence. Pregnant women who had a history of abortion were four times more likely to adhere to Iron and Folic Acid supplementation than their counterparts. This evidence is supported by a study conducted in Maputo, Mozambique [25]. This might be because pregnant women who had a history of abortion may have a fear that abortion can happen again. Consequently, this may encourage them to give more emphasis to their antenatal care and the supplement too. In addition, they might be exposed to health related information during abortion and post abortion care from health care providers in which they might be aware that abortion is caused by Iron deficiency anemia and can be prevented by consumption of IFA tablets as recommended.

Pregnant women who had knowledge about anemia were more likely adherent than those who had no. This result is supported by study reports from Ethiopia and Western Iran [23, 26]. This might be related with the knowledge they had on anemia, the consequences resulted in and the prevention strategy. The current study also found that women who received health education had better adherence than who did not received. Similar findings were reported from Ethiopia [27], Rio de Janeiro [28], Nepal [29] and India [30]. The possible explanation may be that pregnant women who received health education might have the opportunity to understand the purpose, importance, possible side effects and duration and dosage of the supplement.

Another factor that had a significant association with IFA supplementation in the present study was knowledge about the benefit of the supplement. Those pregnant women who were knowledgeable about the benefit of IFA supplement were more likely to be adherent to the supplement. This finding is consistent with other studies conducted in Ethiopia [22, 23], Western Iran [26].

Furthermore, this study revealed that pregnant women who did not face problems in the health institution during collection of the supplement were 4.8 times more likely to adhere to the IFA supplement than those who have faced problems. The finding is supported by studies conducted in Nigeria, India and Nepal [31–33]. The reason might be due to the fact that long waiting time to get the supplement, inadequate supply of IFA tablets in the health facility may hinder adherence of pregnant women with IFA supplementation. Regarding to this evidence further research is required concerning problems in the health facilities.

### Limitation of the study

Since the study was based on the previous one-month intake of Iron and Folic Acid tablets, it might be subjected to potential recall bias. Another limitation of the study might be that Iron and Folic Acid adherence was determined by pregnant women’s response (self-reported adherence measuring method) which might not reflect the actual adherence rate of the source population. In addition, the estimation of Iron and Folic Acid supplementation adherence by self-report method may underestimate the prevalence of non-adherence when compared with objective measures like pill counts or biological assays medication adherence measures. The fact that serum haemoglobin level of pregnant women was not also measured to confirm the diagnosis of anemia in pregnancy can also be another limitation of this study.

## Conclusion

Overall, less than half of the pregnant women had taken Iron and Folic Acid tablets as per the World Health Organization recommendation. This shows that less than an average level of adherence as compared to that reported from different study findings. Health education, knowledge of anemia, history of abortion, knowledge about benefits of Iron and Folic Acid supplementation and not facing problems in the health facilities during ANC follow up were factors statistically significantly associated with adherence to Iron and Folic Acid supplementation among pregnant women attending ANC.

Therefore, health care facilities and providers should strengthen efforts to provide information and create awareness about the importance or benefits of IFA supplementation to both mothers and their foetus. Moreover, the Government of Ethiopia, ministry of health should improve the quality of health facilities in delivering the services and adequate supply of IFA tablets to enhance adherence of pregnant women to IFA supplementation.

## Data Availability

Data will be available upon request from the corresponding author.

## Abbreviations

ANC: Antenatal care
AOR: Adjusted odds ratio
CI: Confidence interval
COR: Crude odds ratio
IFA: Iron and Folic Acid
IFAS: Iron and Folic Acid Supplementation
MMAS (MMAS Version_8): Morskiy medication adherence score
SD: Standard deviation
WHO: World Health Organization
